# DRP1 Inhibition Rescues Mitochondrial Integrity and Excessive Apoptosis in CS-A Disease Cell Models

**DOI:** 10.3390/ijms22137123

**Published:** 2021-07-01

**Authors:** Barbara Pascucci, Francesca Spadaro, Donatella Pietraforte, Chiara De Nuccio, Sergio Visentin, Paola Giglio, Eugenia Dogliotti, Mariarosaria D’Errico

**Affiliations:** 1 Institute of Crystallography, Consiglio Nazionale delle Ricerche, 00015 Rome, Italy; barbara.pascucci@ic.cnr.it; 2 Department of Environment and Health, Istituto Superiore di Sanità, 00161 Rome, Italy; eugenia.dogliotti@iss.it; 3 Core Facilities, Istituto Superiore di Sanità, 00161 Rome, Italy; francesca.spadaro@iss.it (F.S.); donatella.pietraforte@iss.it (D.P.); 4 Research Coordination and Support Service, Istituto Superiore di Sanità, 00161 Rome, Italy; chiara.denuccio@iss.it; 5 National Center for Research and Preclinical and Clinical Evaluation of Drugs, Istituto Superiore di Sanità, 00161 Rome, Italy; sergio.visentin@iss.it; 6 Department of Biology, Tor Vergata University, 00133 Rome, Italy; paola.giglio@uniroma2.it

**Keywords:** cockayne syndrome, mitochondrial dysfunction, apoptosis, MDIVI-1

## Abstract

Cockayne syndrome group A (CS-A) is a rare recessive progeroid disorder characterized by sun sensitivity and neurodevelopmental abnormalities. Cells derived from CS-A patients present as pathological hallmarks excessive oxidative stress, mitochondrial fragmentation and apoptosis associated with hyperactivation of the mitochondrial fission dynamin related protein 1 (DRP1). In this study, by using human cell models we further investigated the interplay between DRP1 and CSA and we determined whether pharmacological or genetic inhibition of DRP1 affects disease progression. Both reactive oxygen and nitrogen species are in excess in CS-A cells and when the mitochondrial translocation of DRP1 is inhibited a reduction of these species is observed together with a recovery of mitochondrial integrity and a significant decrease of apoptosis. This study indicates that the CSA-driven modulation of DRP1 pathway is key to control mitochondrial homeostasis and apoptosis and suggests DRP1 as a potential target in the treatment of CS patients.

## 1. Introduction

Cockayne syndrome (CS) is a multi-system disorder with defects in the transcription coupled nucleotide excision repair (TC-NER) involved in the repair of UV damage from the transcribed strand of active genes. Over 90% of CS cases are due to mutations in either the CSA or CSB genes. CS is cancer-free and the cardinal clinical features are pre- or post-natal growth failure, progressive neurological dysfunction and premature aging. All these symptoms are difficult to trace back to UV damage repair defects only.

The sensitivity of CS cells to oxidatively-generated DNA damage [1,2,3] indicates that CSA and CSB proteins play a role in additional pathways. Accumulation of oxidative DNA damage (both oxidized DNA bases and cyclopurines) in nuclear DNA [2,4,5], elevated levels of mitochondrial DNA damage, hypersensitivity to bioenergetic inhibitors, altered organization of mitochondrial respiratory complexes and redox unbalance attributable to an overproduction of mitochondrial reactive oxygen species (ROS) have been reported in both CS-A and CS-B cells [4,6,7,8,9]. In line with a key role of these proteins at mitochondria, CSA and CSB localize at mitochondrial nucleoids [10,11] where they interact with base excision repair enzymes [10,11] and with proteins involved in mitochondrial transcription [12]. In addition, mitochondrial dynamics is deregulated in CS-A cells that present increased mitochondrial fragmentation and hyperactivation of the fission factor dynamin-related protein DRP1 [13].

DRP1 is present in the cytoplasm and upon activation binds at fission sites on the inner mitochondrial membrane [14]. DRP1 activity is regulated by a series of post-translational changes including phosphorylation, sumoylation, ubiquitination and S-nitrosylation [15]. Excessive activation of DRP1 by phosphorylation or nitrosylation, and therefore of increased mitochondrial fission, can cause neurodegenerative features [16]. In fact, it has been reported that neurodegenerative disorders such as Huntington’s disease (HD), Parkinson’s disease (PD) and Alzheimer’s disease (AD) present fragmented mitochondria with impaired bioenergetics, and this is regarded as the core of their pathological processes [17,18,19].

Here, we used an isogenic CS-A cell system [13] as a model to gain a deeper understanding of the role of DRP1 in CS pathogenesis. We show that DRP1 and CSA are recruited to mitochondria after mitochondrial damage. When enzymatic activity of DRP1 is inhibited, either by chemical inhibition or gene silencing, the dysfunctional mitochondrial and apoptotic phenotype of CS-A cells are recovered. All these data clearly indicate that the modulation of enzymatic activity of DRP1 by post-translational modifications is critical in CS-A cells, suggesting DRP1 as therapeutic target and its inhibitors as potential therapeutic tools.

## 2. Results

### 2.1. DRP1 and CSA Translocate to Mitochondria after CCCP Treatment

CS is characterized by mitochondrial dysfunction [4,7,13]. Two independent studies indicate that CSA protein is present in mitochondrial extracts [10,13]. Moreover, in a previous study we provided evidence of an interaction between DRP1 and CSA in the cytosol, both in basal conditions and after exposure to the mitochondrial uncoupler carbonyl cyanide m-chloro phenyl hydrazone (CCCP) [13]. To further investigate the interaction between CSA and DRP1 we took advantage of an isogenic cell model system, i.e., the SV40-transformed CS-A cell line CS3BE and its derivative CS3BE-wtCSA that stably expresses the wild-type CSA protein tagged at the C-terminus with the Flag and HA epitopes. This cell system has been shown to be a “bona fide” model for the bioenergetics defect (i.e., mitochondrial dysfunction and altered redox status/metabolism) of primary CS-A fibroblasts [13]. The interaction between CSA and DRP1 in mitochondria in response to CCCP treatment was analyzed by confocal laser scanning microscopy (CLSM) in the CS3BE-wt CSA cells.

**Figure 1:**
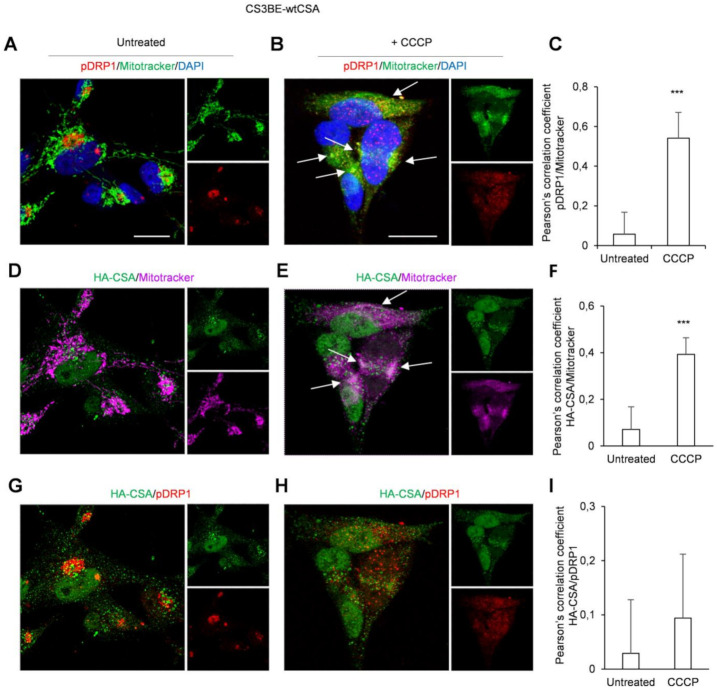
CSA and pDRP1 recruitment to mitochondria after CCCP treatment in CS3BE-wtCSA cells CLSM examinations of CS3BE-wtCSA cells in the absence (**A**,**D**,**G**) or in the presence (**B**,**E**,**H**) of CCCP for 16 h. Living cells were stained with Mitotracker^®^ Deep Red FM (detected in green or magenta), fixed, permeabilized and labelled for pDRP1 (detected in red) and HA-CSA (green) expression. DAPI was used to stain nuclei (blue). Merged images of pDRP1/Mitotracker (**A**,**B**), HA-CSA/Mitotracker (**D**,**E**) and pDRP1/HA-CSA (**G**,**H**) are reported and insets represent separate channel images. Arrows indicate intracellular compartments where colocalizations of reported molecules are observed (colocalizations are detected in yellow or white). Scale bars, 20 µm. Images are representative of three independent experiments. Quantification of colocalization of pDRP1 with mitochondria (**C**) or HA-CSA with mitochondria (**F**) and of HA-CSA with pDRP1 (**I**) after cell treatment with CCCP, as determined by Pearson’s correlation coefficient measured in all microscopy images (mean ± SD). *** *p* < 0.0001, by Mann-Whitney test.

Untreated cells showed detectable levels of the phospho-active form of DRP1 (pDRP1, phosphorylated at Ser 616) outside the mitochondria (Figure 1A), with CSA mainly present inside the nuclei with spots diffused in the cytoplasm (Figure 1D). After treatment with CCCP (20 μM for 16 h), pDRP1 turned into a more punctuate and broad pattern largely overlapping with mitochondria (Figure 1B), where CSA was also recruited (Figure 1E). The unbiased global Pearson’s coefficient confirmed a significant increase in the colocalization of pDRP1 (Figure 1C) and of CSA with mitochondria (Figure 1F) in the CS3BE-wt CSA cells in response to CCCP treatment. However, no mitochondrial superimposition between pDRP1 and CSA was observed by the immunofluorescence analyses (Figure 1G,H), neither revealed by the Pearson’s coefficient (Figure 1I).

In line with previous results [13], the level of pDRP1 in CS3BE cells (Figure S1A) was significantly higher (*p* < 0.05) than in normal cells under basal conditions and also after CCCP treatment, although in this case the difference did not reach statistical significance. Figure S1B,C provide further details about pDRP1 distribution upon CCCP treatment in CS3BE cells, showing that the majority of pDRP1-rich spots colocalize with mitochondrial structures and present a diffuse distribution when compared to untreated cells where they localize in discrete perinuclear regions.

Intriguingly, either in the CS3BE-wtCSA, or in its defective counterpart, pDRP1 signals mostly overlapped the area of the Golgi apparatus—the major transit station where many proteins from the endoplasmatic reticulum are modified and directed to their final destination—while treatment with CCCP induced a redistribution of this protein into other cytoplasmic compartments (Figure S2A,B), most likely mitochondria, as also evidenced by the Pearson’s coefficient (Figure S2C).

Taken together, these results suggest that CSA and pDRP1, after mitochondrial damage are both, but independently, recruited to the mitochondrial compartments.

### 2.2. Inhibition of DRP1 Ameliorates the Dysfunctional Mitochondrial Phenotype of CS-A Cells

One hallmark of CS-A cells is the increased mitochondrial fission due to DRP1 hyperactivation [13]. Mitochondrial fission is crucially regulated by the activity of DRP1 that self-assembles into spirals causing membrane constriction. MDIVI-1 [3-(2,4-Dichloro-5-methoxy-phenyl)-2-thioxo-1H-quinazolin-4-one] is a cell-permeable quinazolinone compound that inhibits the assembly of DRP1 and GTPase DRP1 enzymatic activity in vitro [20]. We have previously reported that the fraction of cells containing mitochondria with an elongated shape (tubular) is significantly higher in normal than in CS-A cells, which, conversely, are enriched in fragmented mitochondria [13]. Therefore, in order to verify if DRP1 inhibition ameliorates mitochondrial morphology and functionality, we treated CS-A cells with MDIVI-1.

**Figure 2:**
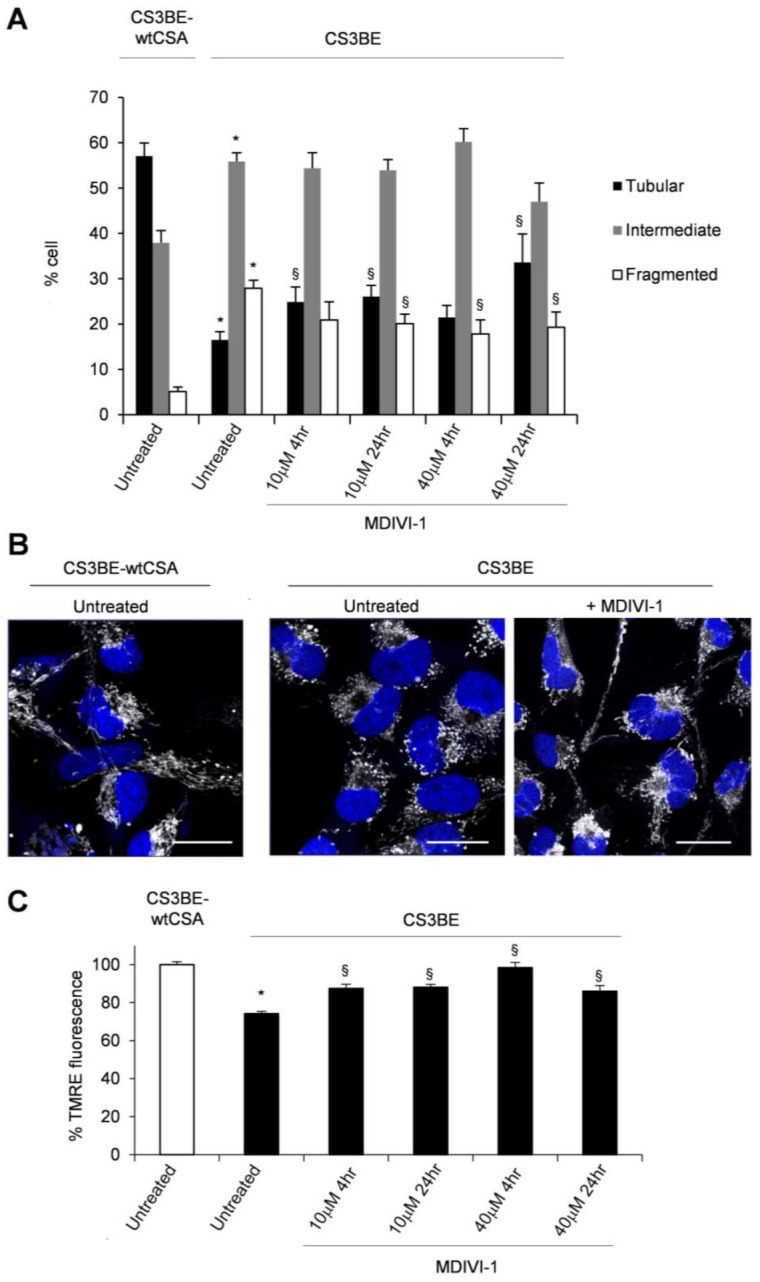
MDIVI-1 treatment ameliorates mitochondrial phenotype in CS3BE cells. (**A**) Distribution of cell percentage according to mitochondrial morphology in CS3BE-wtCSAand CS3BE cells. The reported values represent the mean ±SEM of four independent experiments; * *p* < 0.05 vs. CS3BE-wtCSA cells; § *p* < 0.05 vs. untreated CS3BE cells. (**B**) CLSM examinations (three-dimensional reconstruction images) of CS3BE cells cultured in the presence or absence of MDIVI-1 for 24 h. To detect mitochondria, living cells were stained with Mitotracker^®^ Deep Red FM (pseudo-color gray) and then fixed. Nuclei were stained with DAPI (blue). A representative example of CS3BE-wtCSA cells labelled with Mitotracker^®^ Deep Red FM is reported on the left. Scale bars, 20 µm. Panels are representative of four independent experiments. (**C**) Mitochondrial membrane potential expressed as percentage of TMRE-fluorescence intensity in CS3BE and CS3BE-wtCSA cells. The reported values represent the mean ± SEM of four independent experiments. CS3BE-wtCSA is taken as 100%. * *p* < 0.05 vs. CS3BE-wtCSA cells; § *p* < 0.05 vs. untreated CS3BE cells.

Treatments of CS3BE cells with different concentrations of MDIVI-1 caused a time and a dose dependent increase of cells presenting tubular mitochondria, as revealed by the assessment of mitochondria morphology with the fluorescent probe tetramethylrhodamine, ethyl ester (TMRE) (Figure 2A). In particular, when CS3BE cells were treated with the highest MDIVI-1 dose, 40 uM, for 24 h the fraction of cells containing tubular mitochondria was significantly higher than that detected at the lower drug concentration and following shorter exposure times indicating a progressive recovery of the normal mitochondrial morphology (Figure 2A,B). Then, we asked if beyond the morphological change, there was also an effect on the mitochondrial membrane potential, which is known to be lower in CS3BE as compared to CS3BE-wtCSA cells [13]. After MDIVI-1 treatment, CS-A defective cells showed a statistically significant (*p* < 0.05) recovery of the mitochondrial membrane potential at all doses tested (Figure 2C) as measured by TMRE staining.

**Figure 3:**
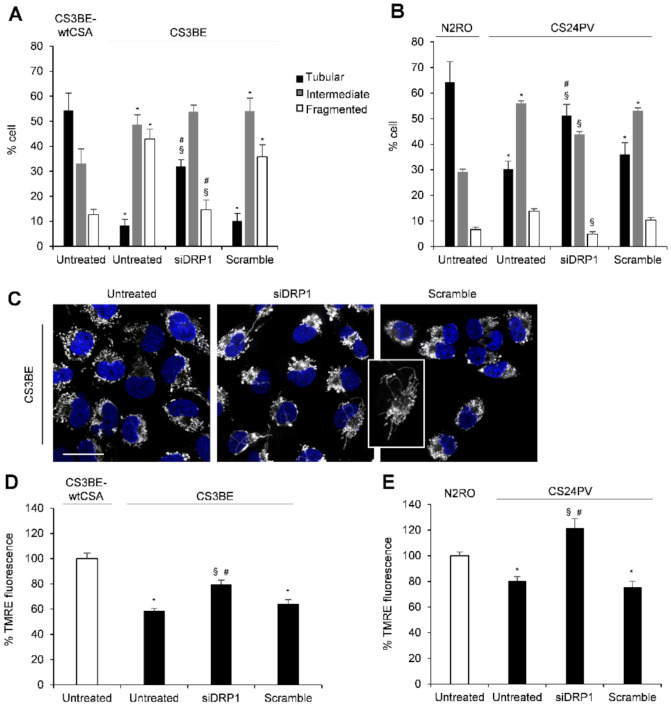
DRP1 silencing ameliorates mitochondrial phenotype in CS3BE cells. (**A**,**B**) Distribution of cell percentage according to mitochondrial morphology in SV40-trasformed cells (CS3BE-wtCSA and CS3BE), and normal (N2RO) and CS-A (CS24PV) primary fibroblasts. The reported values represent the mean ±SEM of four independent experiments; * *p* < 0.05 vs. CS3BE-wtCSA and N2RO cells; § *p* < 0.05 vs. untreated CS3BE or CS24PV cells; # *p* < 0.05 vs. scramble CS3BE or CS24PV cells. (**C**) CLSM examinations (three-dimensional reconstruction images) of CS3BE cells: untreated (left), silenced (middle) and scramble (right). To detect mitochondria, living cells were stained with Mitotracker^®^ Deep Red FM (pseudo-color gray) and then fixed. Nuclei were stained with DAPI (blue). Scale bars, 20 µm. Panels are representative of two independent experiments. (**D**,**E**) Mitochondrial membrane potential expressed as percentage of TMRE-fluorescence intensity in SV40-trasformed cells (CS3BE-wtCSA and CS3BE), and normal (N2RO) and CS-A (CS24PV) primary fibroblasts. The reported values represent the mean ± SEM of four independent experiments. The values obtained in CS3BEwtCSA and N2RO cells are taken as 100%. * *p* < 0.05 vs. CS3BE-wtCSA or N2RO; § *p* < 0.05 vs. untreated CS3BE or CS24PV cells; # *p* < 0.05 vs. scramble CS3BE or CS24PV cells.

The effect of DRP1 inhibition in CS-A cells was further confirmed by genetic silencing of DRP1. In these experiments, besides the SV-40 transformed CS3BE cells, normal (N2RO) and CS-A primary fibroblasts (CS24PV) were used. Transfection with DRP1 siRNA effectively reduced (approximately 85%) DRP1 expression in both CS3BE and CS24PV cells (Figure S3). As shown in Figure 3, siDRP1 CS-A fibroblasts, either established (panel A) or primary (panel B), showed a significant reduction of the fraction of cells with fragmented mitochondria and an increase of those presenting tubular mitochondria, as compared to untreated cells (Figure 3A,B). The mitochondrial morphology profile of the corresponding normal cells, CS3BE and N2RO respectively, is shown for comparison. CLSM examinations of siDRP1 CS3BE cells clearly highlighted the elongated and tangled shape of mitochondria, at variance with the rounded one observed in untreated and scramble cells (Figure 3C). Interestingly, a recovery of the mitochondrial membrane potential, as measured by TMRE was also observed in DRP1-knockdown transformed and primary CS-A cells (Figure 3D,E).

Overall, these data indicate that both pharmacological inhibition and genetic knockdown of DRP1 effectively ameliorated the mitochondrial morphology and membrane potential of CS-A cells.

### 2.3. Reactive Oxygen and Nitrogen Species Are in Excess in CS-A cells and Decrease Ipon DRP1 Inhibition

**Figure 4:**
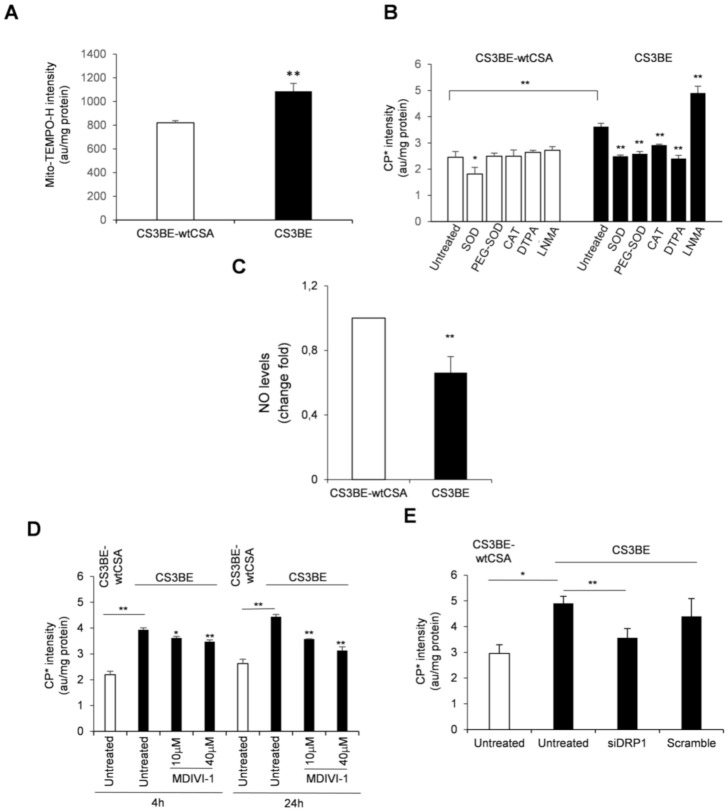
(**A**) Mitochondrial ROS levels assessed by EPR technique by using the cyclic hydroxylamine spin probe Mito-TEMPO-H in CS3BE and CS3BE-wtCSA cells. (**B**) ROS species characterization in CS3BE and CS3BE-wtCSA cells by competition with the suitable scavengers for O2-• and H_2_O_2_ as indicated. SOD, superoxide dismutase; PEG-SOD, superoxide dismutase conjugated to polyethylene glycol (PEG); CAT, catalase; DTPA, diethylenetriamine pentaacetic acid, and NMA, N-monomethyl-L-arginine. (**C**) NO levels in CS3BE and CS3BE-wtCSA cells measured by DAF-FM-DA. D, E. ROS levels assessed by EPR technique by measuring the intensity of the formed nitroxide 3-carboxyproxyl radical in CS3BE and CS3BE-wtCSA cells after treatment with MDIVI-1 (**D**) or DRP1 silencing (**E**). The reported values are mean ± SD of three independent experiments; * *p* < 0.05, ** *p* < 0.01 by paired Student t-test.

It is well established that the absence of CS proteins is associated with overproduction of radical species [4]. Less is known about their characterization. We have previously shown, by using the mitochondrial superoxide indicator mito-SOX, that a relevant portion of ROS, present at high levels in CS-A cells, originates from mitochondria [13]. To support this observation, the cyclic hydroxylamine spin probe Mito-TEMPO-H, a modified nitroxide that is analogous to mito-SOX [21], was used to investigate by EPR spectroscopy the subcellular localization of ROS in CS3BE cells. Mito-TEMPO-H actively accumulates in the mitochondrial matrix providing EPR signals attributable to superoxide generated mainly in this subcellular compartment [21]. Pre-incubation of cells with Mito-TEMPO-H (Figure 4A) showed that, in addition to cytoplasm, also mitochondria are a source of ROS in CS3BE cells (increase of EPR signals of about 32 % when compared to CS3BE-wtCSA cells).

To further characterize the ROS species produced in CS3BE cells, we used the spin probe 1-hydroxy-3-carboxy-2,2,5,5-tetramethylpyrrolidine (CPH). This compound can permeate the cells, and is not specific for a singular oxidant, but suitable to screen the totality of ROS produced in biological samples [21]. We then performed competition experiments by incubating CPH with cells treated with (i) suitable scavengers for O_2_-^•^ and H_2_O_2_, i.e., superoxide dismutase (SOD, 10 µg/mL), its cell permeable form (i.e., conjugated to PEG, PEG-SOD, 10 µg/mL) and catalase (CAT, 10 µg/mL), respectively, (ii) a metal chelating agent (i.e., diethylenetriamine penta-acetic acid, DTPA, 1 mM) and (iii) an inhibitor of nitric oxide (NO) synthase (i.e., N^G^-monomethyl-l-arginine, LNMA, 5 mM). Catalase and DTPA are cell-impermeable compounds, while PEG-SOD reasonably taken up by endocytosis. ROS species were measured by EPR as above. With the exception of SOD, which faintly, but significantly (*p* = 0.03) affected EPR signal intensity in CS3BE-wtCSA cells, the presence of scavengers/inhibitors did not affect ROS formation (Figure 4B). Conversely, in CS3BE cells SOD, PEG-SOD, CAT and DTPA significantly decreased signal intensity, suggesting a superoxide-mediated, metal-dependent ROS formation. Interestingly, incubation with LNMA resulted in increased probe oxidation in CS-A cells, suggesting that NO synthesis inhibition made superoxide more available. Indeed, superoxide and NO are known to react very fast and generate peroxynitrite, whose excess has been previously described in CS defective cells [9]. If high levels of peroxynytrite are formed in CS-A cells this might affect the intracellular bioavailability of NO. This hypothesis was investigated by measuring the levels of NO in normal and CS-A cells by using the probe 4-amino-5-methylamino-2′,7′-difluorofluorescein diacetate (DAF-FM diacetate). This molecule is known to fluoresce proportionally to its binding of NO [22]. The fluorescence measured in CS3BE cells was significantly lower (34%) with respect to CS3BE-wtCSA cells (*p* = 0.009) (Figure 4C), confirming previous EPR data and suggesting that the higher levels of superoxide measured in CS-A cells may be the result of low NO levels.

These data indicate that excessive ROS in CS-A cells were partially of mitochondrial origin and were generated by a superoxide-mediated and metal-dependent process. Moreover, reduced NO levels suggest that the levels of reactive nitrogen species were also altered in CS-A cells.

Excessive fission is associated with ROS overproduction. Therefore, we tested whether the mitochondrial fission inhibitor (MDIVI-1) was able to modulate the excess of intracellular ROS of CS-A cells. Cell treatment with MDIVI-1 for 4 and 24 h at two doses (10 and 40 µM) induced a significant reduction of intracellular ROS levels in CS3BE cells, as measured by electron paramagnetic resonance (EPR) (Figure 4D). The effect was higher at the longer exposure time with the lowest dose of MDIVI-1 (dose at which no cytotoxic effect was observed) reducing the excess of ROS to approximately one third. A significant decrease of ROS level (approaching that of normal cells) was also observed in CS3BE cells upon silencing of DRP1 (Figure 4E).

These results show that inhibition of DRP1 by both MDIVI-1 and DRP1 knockdown was able to reduce the level of intracellular ROS typical of CS-A cells, emphasizing the key role of excessive fission in ROS overproduction.

### 2.4. DRP1 Inhibition Is Able to Reduce the Apoptotic Rate of CS-A Cells

**Figure 5:**
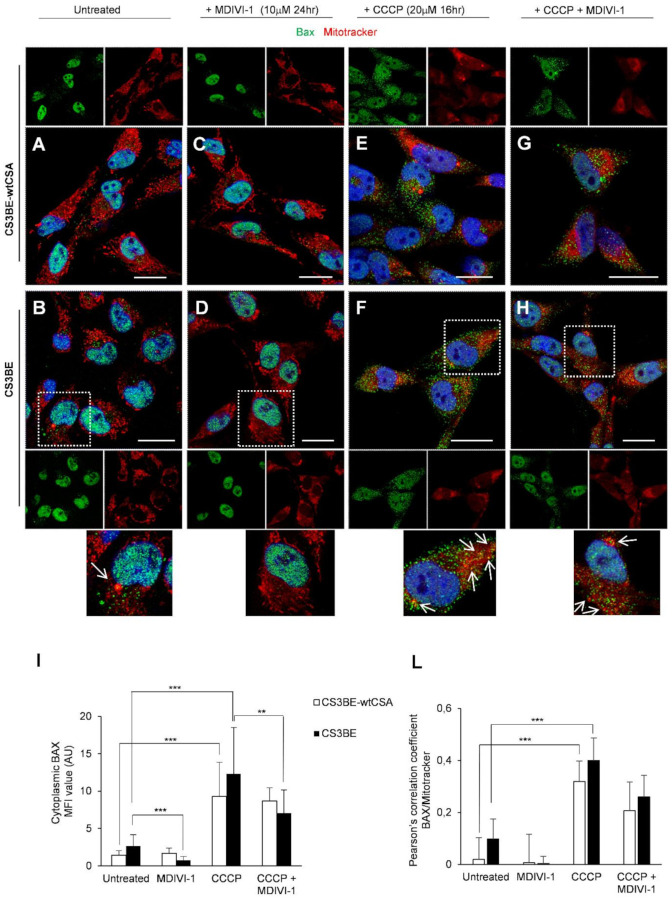
MDIVI-1 treatment decreases the level of apoptotic Bax at mitochondria. (**A**–**H**) Detection of Bax and mitochondria by CLSM examinations in CS3BE-wtCSA and CS3BE cells. (**A**,**B**) untreated; (**C**,**D**) MDIVI-1 (10 µM for 24 h); (**E**,**F**) CCCP (20 µM for 16 h); (**G**,**H**) MDVI-1 and CCCP treated. Living cells were stained with Mitotracker^®^ Deep Red FM (detected in red), fixed, permeabilized and labelled with polyclonal anti-Bax Ab (green). Nuclei are reported in blue (DAPI). Colocalization areas (detected in yellow) are shown in merged images. Insets represent separate channel images. For CS3BE cells a higher-power magnification image of a selected cell for each experimental condition is shown, with arrows indicating Bax-positive mitochondria. Scale bars, 20 µm. Panels are representative of three independent experiments. (**I**). Mean fluorescence value of cytoplasmic Bax in each experimental condition. (**L**). Quantification of colocalization of Bax with mitochondria after treatment with MDIVI-1 and/or CCCP, as determined by Pearson’s correlation coefficient measured in all microscopy images (mean ± SD). *** *p*< 0.0001, ** *p* < 0.01 by Mann–Whitney test.

The phenotypic hallmark of CS cells is increased apoptosis [23,24]. In line with the increased susceptibility of CS-A cells to apoptosis we have previously shown that the number of Bax-positive mitochondria is significantly higher, when CSA is defective, in both SV-40 transformed and primary fibroblasts [13]. We here tested whether the MDIVI-1 treatment, besides rescuing the defective mitochondrial phenotype, was able to inhibit apotosis in CS-A defective cells. Bax is a key regulator of apoptosis that under stress translocates from the cytosol to mitochondria. The level of Bax at mitochondria was monitored by using an antibody specific for its activated oligomeric form that occurred specifically at mitochondria. CS3BE cells were confirmed to contain a higher frequency of Bax-positive mitochondria than the CS3BE-wtCSA cells under basal conditions (Figure 5A,B,I) and MDIVI-1 treatment was able to revert this phenomenon (Figure 5D,I). Upon induction of apoptosis by incubation with the uncoupler CCCP Bax largely colocalized with mitochondria both in CS3BE-wtCSA and CS3BE cells (Figure 5E,F,I,L), and MDIVI-1 significantly reduced the amount of Bax-positive mitochondria CS3BE cells (Figure 5G–L).

**Figure 6:**
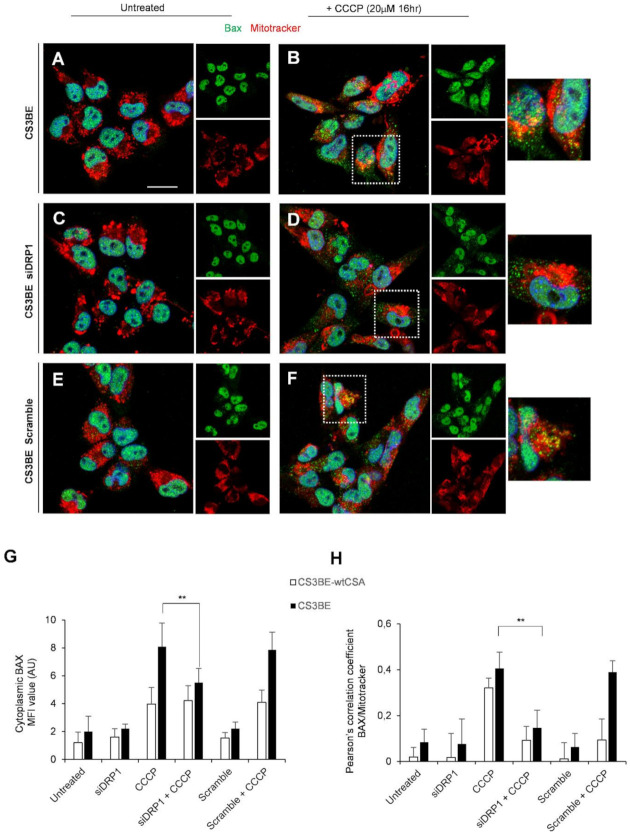
DRP1 silencing decreases the level of apoptotic Bax at mitochondria. (**A**–**F**) Detection of Bax and mitochondria by CLSM examinations in CS3BE cells. (**A**,**C**,**E**) untreated; (**B**,**D**,**F**) CCCP (20 µM for 16 h) treated**; (C**,**D**) after DRP1 silencing; (**E**,**F**) in the presence of Scramble. Living cells were stained with Mitotracker^®^ Deep Red FM (detected in red), fixed, permeabilized and labelled with polyclonal anti-Bax Ab (green). Nuclei are stained in blue (DAPI). Colocalization areas (detected in yellow) are shown in merged images. Insets represent separate channel images. For CCCP treatment a higher-power magnification image of a selected cell for each experimental condition is shown on the right. Scale bars, 20 µm. Panels are representative of two independent experiments. (**G**) Mean fluorescence value of cytoplasmic Bax in each experimental condition. (**H**) Quantification of colocalization of Bax with mitochondria after DRP1 silencing and/or CCCP treatment, as determined by Pearson’s correlation coefficient measured in all microscopy images (mean ± SD). ** *p* < 0.01 by Mann–Whitney test.

In line with these results, genetic knockdown of DRP1 significantly reduced the proportion of Bax colocalizing with mitochondria in CS3BE cells treated with CCCP (Figure 6B,D), as compared to CS3BE cells without DRP1 silencing treated with CCCP (** *p* < 0.01, Figure 6G), as indicated also by Pearson’s coefficient (Figure 6H). We also performed similar experiments with another specific antibody to Bax (against aa 55–178), which reveals intracellular Bax expression only in apoptotic cells. As indicated in Table S1, silencing of DRP1 in CS3BE cells appreciably reduced the relative percentage of apoptotic Bax positive cells in cultures treated with CCCP with respect to control cells (normalized to 1).

All together, these data indicate that inhibition of DRP1, by means of MDIVI-1 treatment or genetic knockdown, displayed an anti-apoptotic function supporting the notion that mitochondrial fission was a prerequisite for Bax cluster formation on mitochondria and consequently strengthening the central role of DRP1 in CSA mitochondrial dysfunction.

## 3. Discussion

In this study, by targeting DRP1 either by pharmacological or genetic knockdown, we show that there is a causal link between DRP1 hyperactivation and CS-A cell metabolic derangement and excessive apoptosis. We propose that modulation of DRP1 phosphorylation by CSA, and possibly CSB, is crucial to maintain mitochondrial homeostasis.

Initially, we asked what is the fate of CSA and DRP1 in mitochondria upon mitochondrial damage. It is well known that CSA (like CSB) localizes to mitochondria where interacts with BER proteins [10] playing a direct role in repair of mitochondrial DNA damage by stabilizing repair complexes at the membrane site [11].

By CLSM experiments, we show that CSA is mainly localized in the nucleus [25,26] with some spots present also in the cytoplasm, but when cells are challenged with a mitochondrial toxin, like CCCP, CSA increases its mitochondrial localization. Similar behavior was observed in normal fibroblasts for the activated form of DRP1. In the absence of mitochondrial damage pDRP1 is primarily present in the Golgi apparatus, but upon mitochondrial injury a massive redistribution of pDRP1 into mitochondria occurs, showing a dotted and diffuse pattern. In a previous paper [13] we demonstrated that wild type CSA interacts with DRP1 in the cytosol both in basal conditions and after incubation with CCCP and when defective leads to DRP1 hyperactivation. On this basis, we hypothesized a role of CSA in the regulation of DRP1 homeostasis, both by affecting its translocation and its turnover at the mitochondrial level. In this study, we show that CSA and DRP1 proteins translocate to mitochondria upon oxidative stress, but do not colocalize, as revealed by immunofluorescence and Pearson’s coefficient, in line with their independent functional roles within damaged mitochondria.

In CS-A cells the fusion–fission imbalance caused by hyperactivation of DRP1 leads to accumulation of fragmented mitochondria that is not counterbalanced by increased mitophagy ([13] and this study). Alteration of mitochondrial dynamics has been reported in many different neurodegenerative diseases, suggesting common underlying pathways behind mitochondrial dysfunction and neuronal damage. In particular, increased DRP1 expression and mitochondrial fragmentation are early and key events in a wide range of neurodegenerative disorders, including AD, HD and PD [27]. Given the importance of a correct mitochondrial dynamics for the functionality of the nervous system, we tested in our model cell type whether the inhibition of DRP1 hyperactivation may be a therapeutic strategy to reduce oxidative stress, mitochondrial fragmentation and possibly neuronal damage in CS patients. To this aim both pharmacological inhibition of DRP1 by MDIVI-1 [20] and genetic knockdown were used.

MDIVI-1, a cell permeable small molecule, was first identified as an inhibitor of mitochondrial division in yeast screens of chemicals. Most evidence indicates that the effects of MDIVI-1 on mitochondrial fission are due to inhibition of the evolutionarily conserved mitochondrial fission GTPase DRP1. However, an effect of MDVI-1 on inhibition of complex 1 of the electron transport chain was also reported [28]. Because of the pleiotropic effects of MDIVI-1, to strengthen the role of DRP1 in the maintenance of mitochondrial integrity and correct cell death programming all the experiments were also carried out following genetic knockdown of DRP1.

When CS defective cells were treated with MDIVI-1 we observed a decrease in the level of ROS, an increase in the frequency of elongated mitochondria and a reduction of the fragmented forms. The same effects were observed upon DRP1 knockdown. We confirm that a relevant source of these ROS is mitochondrial [13] and further characterize these oxidizing species. We show that, besides O_2_-^•^, high levels of H_2_O_2_ are present. This is likely due to the reduced activity of catalase thus exposing CS-A cell organelles and tissues to damage by peroxide. A metal-dependent ROS formation is also identified. XPA defective cells, that present a similar ROS profile, are characterized by increased levels of intracellular iron that might promote ROS generation via Fenton reaction [29,30]. This is not the case of CS-A cells that present normal intracellular iron levels [unpublished observation]. In addition, we provide evidence of increased levels of reactive nitrogen species and decreased levels of NO, in line with previous findings of increased levels of peroxynitrite in CS-A defective cells [9]. It is of interest that O_2_-^•^ scavengers have been recently shown to prevent phosphorylation of DRP1 and its mitochondrial translocation [31]. Superoxide together with H_2_O_2_ are also the dominant radical species present in CS-A cells indicating that the overproduction of these radical species is key for disease progression.

**Figure 7:**
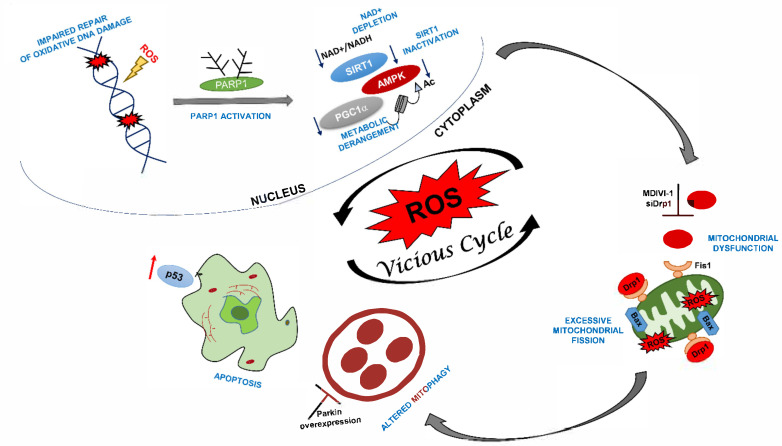
Schematic illustration of proposed mechanisms of neurodegeneration in CS-A patients.

Besides intracellular reactive oxidizing species reduction, treatments with both MDIV-1 and DRP1 siRNA recovered the mitochondrial membrane potential. This is crucial for cell viability and protection from cell necrosis and apoptosis [32]. Coherently, we observed a significant anti-apoptotic effect, as testified by reduced expression of activated Bax at mitochondria. Increased apoptosis ([23], reviewed in [24]), and senescence [33,34] are hallmarks of CS cells that are also characterized by increased levels of p53 ([23], reviewed in [24]). P53 is a key regulator of both apoptosis and senescence and, under cellular stress, translocates to mitochondria by a DRP1-dependent process [35]. Whether deregulated levels of DRP1 and p53 together with the loss of CS proteins represent a convergence of antiproliferative processes deserve further investigation. It is well established that in response to stress the activated sensor protein AMPK phosphorylates the mitochondrial receptor protein MFF, which then recruits DRP1 to the membrane [36]. It has been proposed that modulation of the AMPK-ULK1/DRP1 pathway by CSA and CSB proteins may control this response that is crucial to maintain mitochondrial homeostasis [37]. Our data are in line with this model, by showing a causal link between modulation of DRP1 activation by CSA and regulation of metabolism, mitochondrial health and apoptosis. We propose that the dual role of CS proteins in both repair of oxidatively-induced DNA lesions and modulation of metabolic pathways activated by chronic oxidative stress/energy failure (e.g., PARP1/NAD+/SIRT1/PGC1α axis) [38] converge into mitochondrial dysfunction that is a prominent feature of neurodegeneration onset and progression (Figure 7).

In the absence of CSA the level of intracellular radical species increases dramatically and oxidative DNA damage accumulates in nuclear DNA. Consequently, PARP1 is activated, leading to NAD+ depletion and inactivation of SIRT1 activity, which in turn, influence protein targets involved in metabolic regulation including PGC1α and AMPK (38). The activation of this stress-related axis leads to mitochondrial dysfunction, excessive fission, altered mitophagy and increased apoptosis. These mechanisms severely impact on neuronal health and may progressively lead to neurodegeneration. Inhibition of mitochondrial fragmentation by DRP1 inhibition (this study) or mitophagy by targeting Parkin overexpression [13] helps restoring mitochondrial health, thereby breaking the vicious loop caused by overproduction of ROS.

The successful use in CS-A cells of MDIVI-1 adds to the positive evidence obtained in other “in vitro” and “in vivo” models. In cells, MDIVI-1 retards apoptosis by inhibiting mitochondrial outer membrane permeabilization and blocks Bid-activated Bax/Bak-dependent cytochrome c release from isolated mitochondria [20]. This molecule has been shown to protect hippocampal rat neuronal stem cells [39,40], as well as AD [41,42] and HD [43] model cell systems against oxidative stress, by both preserving mitochondrial integrity and inhibiting mitochondrial apoptotic cascade. Moreover, treatment of ischemia/reperfusion mice with MDIVI-1 blocked apoptotic death of cells involved in cerebral ischemia and reperfusion injury [44].

Because of the pleiotropic role of CS proteins, various therapeutic strategies have been proposed to correct the molecular defects underlying the progressive neural degeneration of these patients, e.g., (i) the inhibition of accumulated mitochondrial serine proteases to restore mitochondrial DNA polymerase-γ levels [9]; (ii) the reduction of endoplasmatic reticulum stress by chemical chaperones to rescue RNA polymerase I activity and protein synthesis [45]; (iii) anti-TNFα therapeutic approaches to prevent apoptosis [46,47] and delay the accelerated aging phenotype [48]; (iv) a ketogenic diet and NAD+ supplementation to stimulate mitochondrial biogenesis [37,38] and (v) HDAC inhibitors to improve autophagic function [49].

In this paper, we achieve a significant mitigation of CS-A cell mitochondrial dysfunction and apoptotic rate by targeting the fission protein DRP1 by both MDIVI-1 and genetic knockdown. In a previous study [13] we obtained a rescue of the dysfunctional mitochondrial phenotype and reduced apoptosis by acting downstream of the accumulation of fragmented mitochondria generated by DRP1 hyperactivation, by overexpressing Parkin, which was able to potentiate the mitophagic process. The use of pharmacological inhibitors is a strategy of election to control deregulated cellular processes rather than manipulating the same biological process by depletion or overexpression of some major players. In conclusion, our findings argue in favor of the bioactivity of MDIVI-1 as an inhibitor of DRP1 mediated fission and support its use as a promising neuroprotective drug for CS patients.

## 4. Materials and Methods

### 4.1. Cell Cultures and Treatment Conditions

Experiments were performed in the SV40-transformed CS-A cell line CS3BE and in its derivative CS3BE-wtCSA that stably expresses the wild-type CSA protein tagged at the C-terminus with the Flag and HA epitopes in the (CS3BE-wtCS-A). SV40-transformed cell lines were cultured as previously described [13,50]. Primary fibroblasts obtained from normal (N2RO) and CSA (CS24PV) donors were also used (these primary fibroblasts were a kind gift from Dott Zambruno, IDI, Rome and Dott Stefanini, CNR, Pavia). Treatments with the mitochondrial uncoupler carbonyl cyanide m-chlorophenylhydrazone (CCCP, 20 μM) (were performed in DMEM with 10% serum for 16 h. Treatments with the mitochondrial fission inhibitor MDIVI-1 were performed in DMEM with 10% serum at different doses and for different times, as reported in the legends to figures.

### 4.2. Small Interfering RNA (siRNA) Treatment of CS Cells

For siRNA treatment, cells were grown to ~50% confluence and then transfected using INTERFERinTM (Polyplus) and 10 nM validated siRNAs against DRP1 (Dharmacon, Lafayette, CO, USA). Gene knockdown was assessed after 72 h using qRT-PCR. For each experiment there was a scrambled siRNA control (Dharmacon). siDRP1 transfection efficiency was assessed by qPCR.

### 4.3. Confocal Laser Scanning Microscopy (CLSM) Analyses

For CLSM analyses, cells were seeded in 24-well cluster plates onto 12-mm cover glasses. After 48 h of culture in complete medium, cells were treated with CCCP and/or MDIVI-1 for 16 h. To detect mitochondria, living cells were stained with Mitotracker^®^ Deep Red FM (250 nM, Thermo Fisher Scientific, (Waltham, MA, USA) for 45′ at 37 °C in serum-free medium. After washing with PBS, cells were fixed by paraformaldehyde 3% (30′, 4 °C), permeabilized by Triton X-100 (0.5% 10′ at room temperature, Sigma-Aldrich) and then stained at 37 °C with different combination of primary Abs: rabbit anti pDRP1 (Cell Signaling) or anti-Bax (Santa Cruz, CA, USA), monoclonal anti-HA (Sigma-Aldrich, St. Louis, MO, USA), anti-Bax (BD) or anti-Golgin-97 Abs (Thermo Fisher Scientific). Alexa Fluor 488- and 594-conjugated secondary Abs were from Thermo Fisher Scientific. The cover glasses were extensively washed with PBS and mounted on the microscope slide with Vectashield antifade mounting medium containing DAPI (Vector Laboratories, Burlingame, CA, USA). CLSM observations were performed with a Leica TCS SP2 AOBS apparatus or with a Zeiss LSM980 microscope, using a 63x/1.40 NA oil objective and excitation spectral laser lines at 405, 488, 594 and 639 nm. Image acquisition and processing were carried out using the Leica Confocal Software 2.6 rel 1537 (Leica Microsystem, Wetzlar, Germany) or the Zeiss Confocal Software Zen 3.1 (Blue edition, Zeiss, Oberkochen, Germany), and Adobe Photoshop CS5 (Adobe Systems Inc, San Jose, CA, USA). Signals from different fluorescent probes were taken in sequential scan settings (three-dimensional reconstruction images) and colocalizations were visualized in merged images. Several cells for each labeling condition were analyzed and representative results are shown. All colocalization analyses were carried out using Image J software (National Institutes of Health, Bethesda, MD, USA) to calculate Pearson’s correlation coefficients. This software estimates the degree of overlap between fluorescence signals obtained in two separate fluorescent channels. The Pearson’s coefficients were calculated from multiple images. Statistical analysis was conducted using GraphPad Prism software (San Diego, CA, USA) and the values represent mean ±SD. Graphs comparing two conditions were analyzed via unpaired t test (Mann–Whitney nonparametric test) and those comparing more than two conditions were analyzed via one-way ANOVA.

### 4.4. Fluorescence Video-Imaging Analysis of Mitochondria Parameters

The potentiometric dye tetramethylrhodamine ethyl ester perchlorate (TMRE) was used to measure mitochondrial membrane potential and to analyze mitochondrial morphology (final concentration 30 nM from 1 mM stock solution in DMSO). In order to reach saturation of the dye, cultured cells were kept for 30′ in the presence of TMRE before recording. To avoid the decay of the signal TMRE was maintained throughout the entire experiment. The composition of the saline solution used for loading and recording was the following (mM): NaCl 140, KCl 5, CaCl_2_ 2.5, MgCl_2_ 1, D-glucose 10, HEPES/NaOH 10 (RT, pH 7.4, 290 mosmol L^−1^). An inverted microscope (Axiovert 135, Zeiss, Oberkochen, Germany) with an oil immersion objective (40×, 1.35 NA, Olympus, Tokyo, Japan) was utilized for fluorescence video imaging. The excitation wavelength of 535 nm was applied by means of a monochromator (Till Photonics, Polychrome II; Munich, Germany). The emission wavelength filtered at 590 nM was collected by a CCD, cooled digital camera (PCO, Sensicam, Kelheim, Germany) and recorded on the HD of a PC computer. The software package Imaging Workbench 6.0 (Indec BioSystems, Santa Clara, CA, USA) was used for recording and off-line analysis of the data. The software allowed the measurement of the emission values also along line profiles crossing mitochondria. To calculate the average amplitude of a given mitochondrion a minimum of two peaks of amplitude was used. Single mitochondria were detectable only in the periphery of a cell, hence, only mitochondria in this area were chosen for analysis. The cells were classified into different groups based on mitochondrial morphology as previously described [13].

### 4.5. Electron Paramagnetic Resonance (EPR) Measurement of ROS Levels

Reactive oxidizing species and RNS detection was performed in 100 µL suspensions of transformed fibroblasts (20 × 10^6^ cells/mL) in PBS, pH 7.4. After the addition of 0.5 mM of the spin probe 1-hydroxy-3-carboxy-2,2,5,5-tetramethylpyrrolidine (CPH; ENZO Life Sciences Inc., NewYork, NY, USA) dissolved in degassed phosphate buffer (PBS; Sigma), pH 7.4, deprived of metal contamination as reported in [4,13] samples were drawn up into a gas-permeable teflon tube with 0.81 mm internal diameter and 0.05 mm wall thickness (Zeuss Industrial Products, Raritan, NJ, Germany). The Teflon tube was folded two times, inserted into a quartz tube, and fixed to the cavity (4108 TMH) of a Bruker ECS 106 EPR spectrometer equipped with a variable temperature unit (ER4111VT). Spectra were acquired 20′ after the addition of the spin probe at 37 °C. The ROS-dependent oxidation of CPH was monitored by the formation of the characteristic three-line spectrum with hyperfine coupling constant of 1.63 ± 0.04 mT attributable to the corresponding nitroxide radical 3-carboxyproxyl radical (CP^•^). The characterization of ROS was performed by pre-incubating cell suspensions with the suitable scavengers, with the inhibitor of nitric oxide synthase or with the metal chelating agent (15′, 37 °C) before CPH addition. It should be noted that since the used scavengers and chelating compounds, with the exception of PEG-SOD, do not cross the plasma membrane, the measurements relate to oxidant species that are released in the extracellular compartment. The investigation of the effects of MDIVI was performed by incubating cells, in DMEM with 10% serum for 4 and 24 h at 37 °C, at the different doses reported in the text. Cells were then recovered, washed, suspended in PBS and processed for EPR measurements by adding CPH, as reported above.

For the measurement of mitochondrial reactive oxidizing species formation, 100 µL of cell suspensions (20 × 10^6^ cells/mL in PBS, pH 7.4) were incubated with 0.5 mM Mito-TEMPO-H for 20 min at 37 °C, then rapidly washed two times in PBS, resuspended in 100 µL PBS, drawn up into the gas-permeable teflon tube and processed as reported above. Spectrometer settings were as follows: modulation frequency, 100 kHz; microwave frequency, 9.6 GHz; microwave power, 10 mW for CPH and 2 mW for Mito-TEMPO-H; modulation amplitude, 0.1 mT for CPH and 0.5 mT for Mito-TEMPO-H; conversion time, 82 ms; time constant, 82 msec for CPH and 164 msec for Mito-TEMPO-H; swep time, 84 s; number of scans, 3 for CPH and 12 for Mito-TEMPO-H.

### 4.6. NO Detection by DAF-FM Staining

Endogenous NO production was assessed in WT and CS-A defective cells 24 h after plating by 30′ incubation with 4-Amino-5-methylamino-2′,7′-difluorofluorescein diacetate (DAF-FM-DA) as previously reported [51]. Fluorescence intensity was analyzed by flow cytometry.

## Data Availability

All data are available from the authors on request.

## Supplementary Materials

The following are available online at https://www.mdpi.com/article/10.3390/ijms22137123/s1.
