# Supplementary material for: DRP1 Inhibition Rescues Mitochondrial Integrity and Excessive Apoptosis in CS-A Disease Cell Models
Source: Int J Mol Sci. 2021 Jul 1;22(13):7123. doi: 10.3390/ijms22137123 (PMC8268695; doi:10.3390/ijms22137123)
Supplement: Supplementary file 1 [file ijms-22-07123-s001.zip › ijms-1273942-supplementary.pdf]

International Journal of *Molecular Sciences*

MDPI

Article

LEGENDS TO SUPPLEMENTARY FIGURES

Figure S1

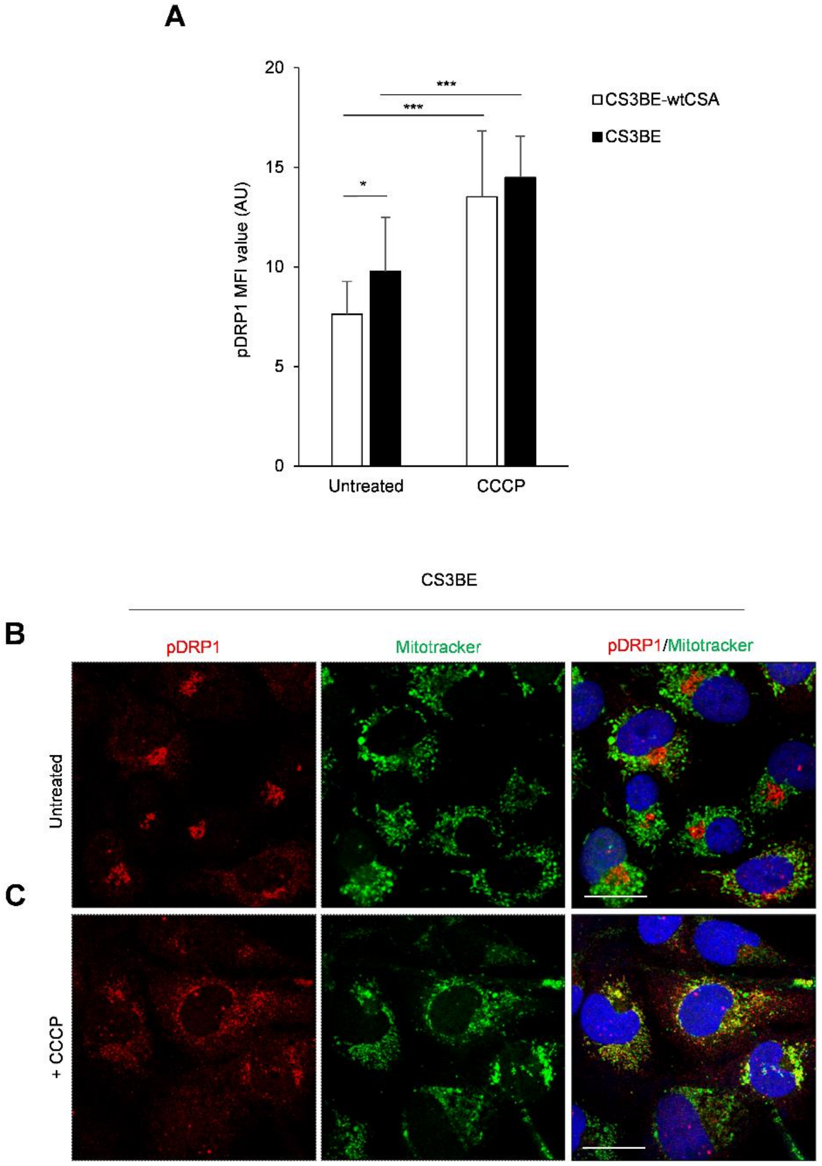

Figure S1. pDRP1 localization at mitochondria in CS-A cells. a. Mean fluorescence intensity value (MFI) of pDRP1 in CS3BE and CS3BE-wtCSA cells after treatment with CCCP. *$p< 0.05$; ***$p< 0.0001$ by Mann-Whitney test. b,c. Detection of pDRP1 and mitochondria by CLSM analysis in CS3BE cells in the absence (b) or in the presence (c) of CCCP for 16h. Living cells were stained with Mitotracker® Deep Red FM (detected in green), fixed, permeabilized and labelled with anti-pDRP1 Abs (detected in red), followed by Alexa Fluor®-594 secondary Abs. Nuclei are reported in blue (DAPI). Colocalization of pDRP1 and Mitotracker (detected in yellow) is showed in merged images. Insets represent separate channel images. Scale bars, 20μm. Representative examples of three independent experiments are shown.

Figure S2

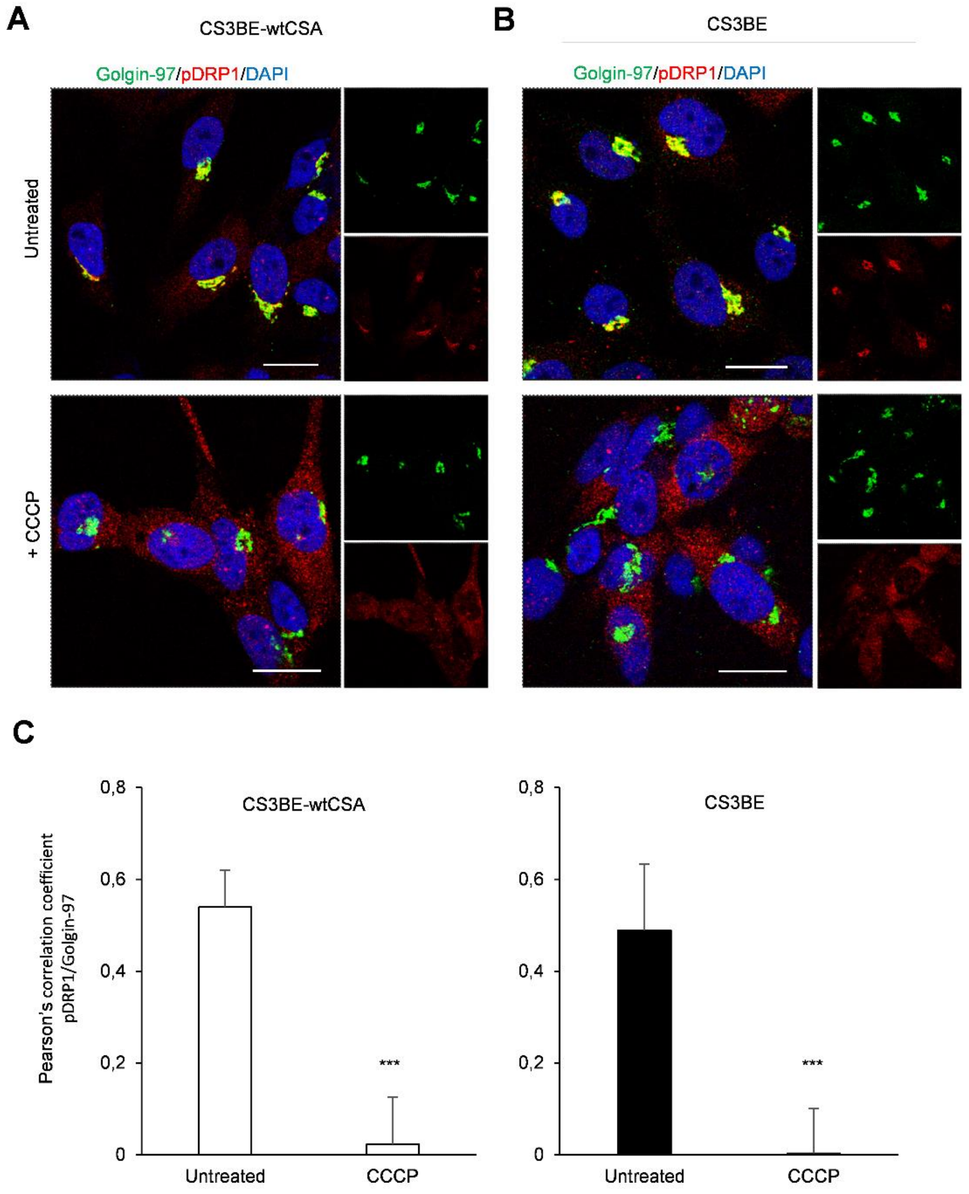

Figure S2. Effect of CCCP treatment on intracellular distribution of pDRP1 in CS3BE-wtCSA and CS3BE cells.CLSM examinations of CS3BE-wtCSA (a) and CS3BE cells (b) untreated or CCCP CCCP for 16h. Cells were fixed, permeabilized and stained with anti-pDRP1 (red) and anti-Golgin-97 Abs (green), followed by secondary Abs. Nuclei are stained in blue (DAPI). Colocaliza-tion between pDRP1 and Golgin protein (detected in yellow) is showed in merged images. Insets represent separate channel images. Scale bars, 20μm. Data are representative of three independent experiments. c. Quantification of colocalization of pDRP1 with Golgi apparatus in the absence or presence of CCCP, as determined by Pearson's correlation coefficient measured in all microscopy images (mean ± SD). ***p< 0.0001 by Mann-Whitney test.

Table S1: Relative fold increase of Bax positive CS3BE-wtCSA and CS3BE cells treated with CCCP versus untreated cells.

		CS3BE-wtCSA	CS3BE	*p* (CS3BE)
**Controll Cell**	Untreated	1	1	
	CCCP	2,47x ±2.26	5.50x ±3.11	<0.0001
**siDRP1**	Untreated	1	1	
	CCCP	2,23x ±2,11	2,18x ±0.90	<0.01
**Scramble**	Untreated	1	1	
	CCCP	2,20x ±1,45	3.95x ±1,40	<0.0001

The reported values represent the relative mean percentage ±SD of two independent experiments. The number of Bax positive cells per field of view was counted under the microscope and cell percentage from CCCP treated cells was related to their control. p calculated by Mann-Whitney test.

Figure S3

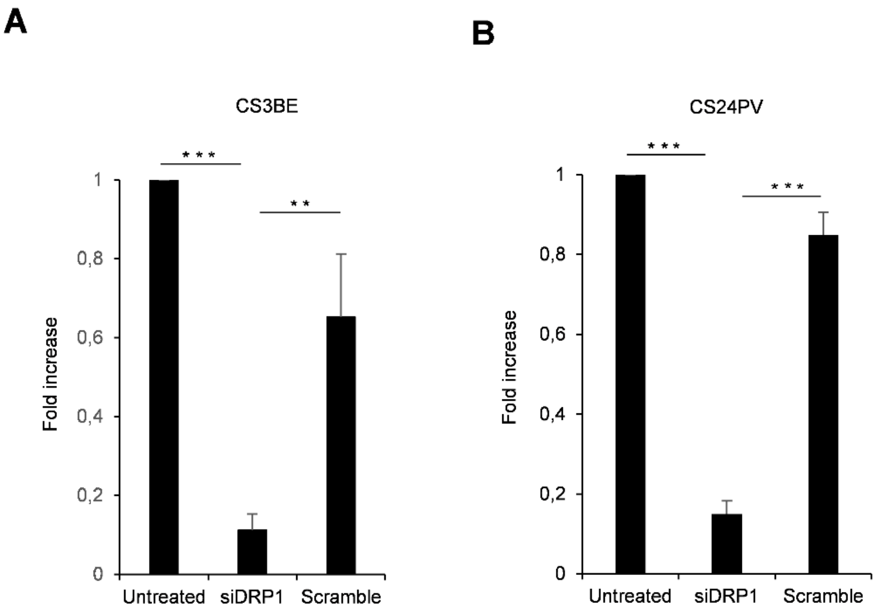

Figure S3. siDRP1 transfection efficiency in CS-A defective cells. Analysis by real time PCR of mRNA levels of DRP1 after silencing in CS3BE and CS24PV cells. ***p< 0.0001, **p< 0.01 by paired Student t-test.
